# Non-contiguous finished genome sequence and description of *Anaerococcus provenciensis* sp. nov.

**DOI:** 10.4056/sigs.5501035

**Published:** 2014-04-10

**Authors:** Isabelle Pagnier, Olivier Croce, Catherine Robert, Didier Raoult, Bernard La Scola

**Affiliations:** 1 Unité de Recherche sur les Maladies Infectieuses et Tropicales Emergentes, UMR CNRS 6236 – IRD 198, Faculté de médecine, Aix-Marseille Université, 27 Boulevard Jean Moulin, 13385 Marseille cedex 05, France

**Keywords:** *Anaerococcus provenciensis*, genome

## Abstract

*Anaerococcus provenciensis* strain 9402080^T^ sp. nov. is the type strain of *A. provenciensis* sp. nov., a new species within the genus *Anaerococcus*. This strain was isolated from a cervical abscess sample. *A. provenciensis* is a Gram-positive anaerobic cocci. Here, we describe the features of this organism, together with the complete genome sequence and annotation. The 2.26 Mbp long genome contains 2099 protein-coding and 57 RNA genes including 8 rRNA genes and exhibits a G+C content of 33.48%.

## Introduction

*Anaerococcus provenciensis* strain 9402080^T^ (= CSUR P121 = DSM 26345) is the type strain of *A. provenciensis* sp. nov. This bacterium is a Gram-positive, non spore-forming, indole negative, anaerobic and non-motile cocci, that was isolated from a cervical abscess sample, during a study prospecting anaerobic isolates from deep samples [1]. Currently, to classify prokaryotes, a polyphasic approach is preferred, combining phenotypic and genotypic characteristics to describe a new isolate [2]. It was recently proposed to integrate genomic features in the description of new bacterial species, because, as a result of decreasing of genomic sequencing costs, more than 3,000 bacterial genome have been sequenced to date [3] providing much information [4-15].

The genus *Anaerococcus* belongs to the order *Clostridiales*, and the family *Clostridiales* Family XI Incertae Sedis [16]. This is a heterogeneous family, grouping anaerobic cocci and rods, and it is mainly defined on the basis of phylogenetic analyses of 16S rRNA gene sequences. Actually, 11 genera are found in the group *Clostridiales* Family XI Incertae Sedis, among which are the genera *Anaerococcus* and *Peptoniphilus.* The genus *Anaerococcus* was first described in 2001 [17], and contains 7 species, *A. prevotii*, *A. hydrogenalis*, *A. lactolyticus*, *A. murdochii*, *A. octavius*, *A. tetradius* and *A. vaginalis*. The type species is *A. prevotii* (type strain ATCC 9321). It was first described in 1948 by Foubert and Douglas [18]. Members of the genus *Anaerococcus* are anaerobic Gram-positive non motile cocci, and formerly belonged to the genus *Peptostreptococcus sp.* bubt were reclassified in 2001 by Ezaki et al., based on phylogenetic and metabolic features [17]. They are mostly found in human vagina, and can also be found in nasal cavity or skin. They have also been implicated in human pathology, and were isolated from several infectious site, such as ovarian, peritoneal, sacral, digital and cervical abscesses, vaginoses, bacteremias, foot ulcers, a sternal wound, and an arthritic knee [17,19-22]. Moreover, uncultured *Anaerococcus sp.* can be detected in metagenomes from the human skin flora [23].

The two species most closely related to *Anaerococcus provenciensis* sp. nov, are *Anaerococcus prevotii* and *Anaerococcus tetradius*, based on the comparison of their 16S rRNA gene sequence.

Here we present a summary classification and a set of features for *A. provenciensis* sp. nov. strain 9402080^T^ (= CSUR P121 = DSM 26345), together with a description of the complete genomic sequencing and annotation. These characteristics support the circumscription of the *A. provenciensis* species.

## Classification and features

A cervical abscess sample was collected from a patient during a study designed to prospect for emerging anaerobes using MALDI-TOF and 16S rRNA gene sequencing, in Marseille [1]. The specimen was preserved at -80°C after sampling. Strain 9402080^T^ (Table 1) was isolated in April 2009 by cultivation on 5% sheep blood-enriched Columbia agar (BioMerieux, Marcy l’Etoile, France), under anaerobic conditions.

**Table 1:** Classification and general features of *Anaerococcus provenciensis* strain 9402080^T^ according to the MIGS recommendations [24]

**MIGS ID**	**Property**	**Term**	**Evidence code^a^**
		Domain *Bacteria*	TAS [25]
		Phylum *Firmicutes*	TAS [26-28]
		Class *Clostridia*	TAS [29,30]
	Current classification	Order *Clostridiales*	TAS [31,32]
		Family XI Incertae Sedis	TAS [16]
		Genus *Anaerococcus*	TAS [17]
		Species *Anaerococcus provenciensis*	IDA
		Type strain 9402080^T^	IDA
	Gram stain	positive	IDA
	Cell shape	cocci	IDA
	Motility	Non-motile	IDA
	Sporulation	Non-sporulating	IDA
	Temperature range	mesophile	IDA
	Optimum temperature	37°C	IDA
MIGS-6.3	Salinity	Weak growth in BHI medium 5% NaCl	IDA
MIGS-22	Oxygen requirement	anaerobic	IDA
	Carbon source	unknown	
	Energy source	unknown	
MIGS-6	Habitat	human	IDA
MIGS-15	Biotic relationship	free living	IDA
MIGS-14	Pathogenicity Biosafety level Isolation	unknown 2 Cervical abscess	
MIGS-4	Geographic location	France	IDA
MIGS-5	Sample collection time	April 2009	IDA
MIGS-4.1	Latitude Longitude	43.296482 5.36978	IDA IDA
MIGS-4.3	Depth	surface	IDA
MIGS-4.4	Altitude	0 m above sea level	IDA

**^a^**Evidence codes - IDA: Inferred from Direct Assay; TAS: Traceable Author Statement (i.e., a direct report exists in the literature); NAS: Non-traceable Author Statement (i.e., not directly observed for the living, isolated sample, but based on a generally accepted property for the species, or anecdotal evidence). These evidence codes are from the Gene Ontology project [33]. If the evidence is IDA, then the property was directly observed for a live isolate by one of the authors or an expert mentioned in the acknowledgements.

**Figure 1:**
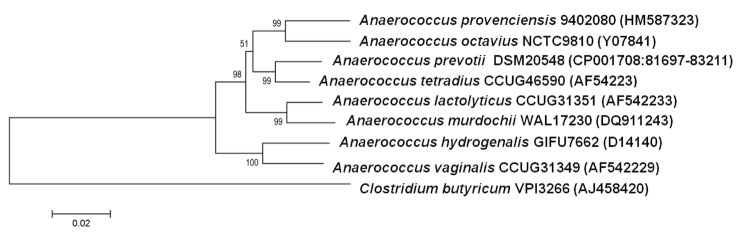
Phylogenetic tree showing the position of *Anaerococcus provenciensis* strain 9402080^T^ relative to other type strains within the genus *Anaerococcus.* GenBank accession numbers are indicated in parentheses. Sequences were aligned using CLUSTALW, and phylogenetic inferences obtained using the maximum-likelihood method within the MEGA 4 software [34]. Numbers at the nodes are bootstrap values obtained from 500 replicates used to generate a majority consensus tree. *Clostridium butyricum* was used as the outgroup. The scale bar represents a 2% nucleotide sequence divergence.

This strain exhibited the highest 16S rDNA nucleotide sequence similarities with a number of *Anaerococcus* species, including *A. octavius* (96%), *A. prevotii* (95%), *A. tetradius* (95%), *A. lactolyticus* (94%), *A. vaginalis* (93%), and *A. hydrogenalis* (93%) (Figure 1). These values are lower than the 98.7% 16S rRNA gene sequence threshold recommended by Stackebrandt and Ebers to delineate a new species without carrying out DNA-DNA hybridization [35].

Seven different growth temperatures (23°C, 25°C, 28°C, 32°C, 35°C, 37°C, 50°C) were tested ; no growth occurred at 50°C; growth occurred in 3 days between 23° and 37°C and optimal growth was observed in 2 days at 35°C and 37°C.

**Figure 2:**
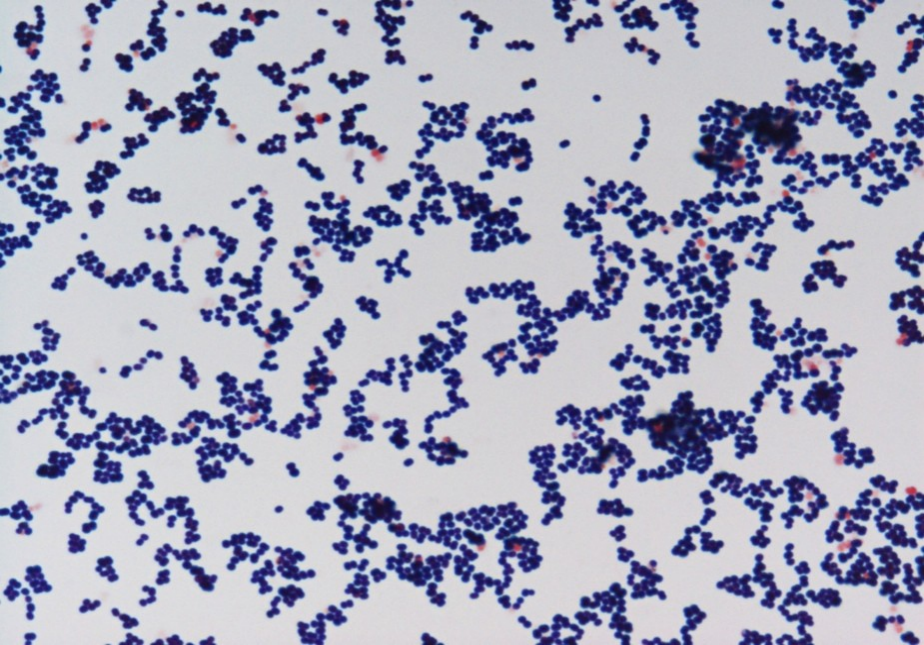
Gram stain of *A. provenciensis* strain 9402080^T^

**Figure 3:**
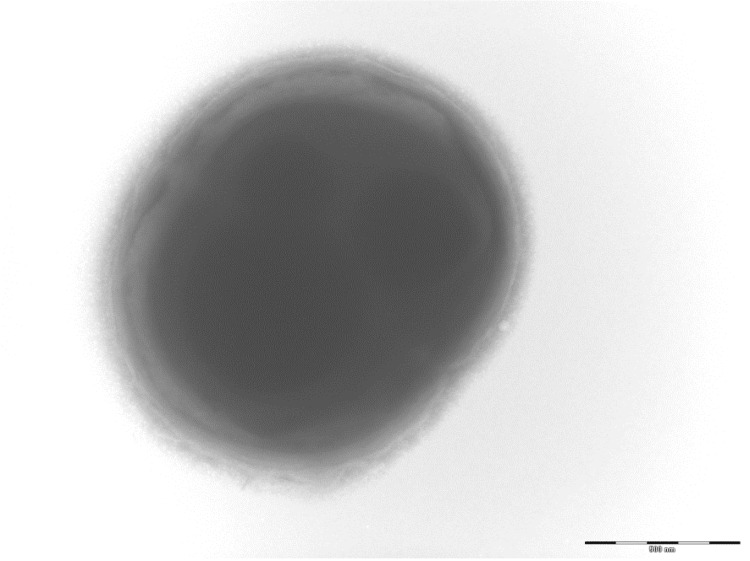
Transmission electron micrograph of *A. provenciensis* strain 9402080^T^, using a Morgani 268D (Philips) at an operating voltage of 60kV. The scale bar represents 1 µm.

Colonies are small, 1mm in diameter, light grey, smooth and round on blood-enriched Columbia agar under anaerobic conditions using GENbag anaer (BioMérieux). Bacteria were grown on blood-enriched Columbia agar (Biomerieux), on BHI agar medium, on BHI agar medium supplemented with 1% NaCl, in BHI broth medium and in Trypticase-soja TS broth medium. Agar plates were incubated under anaerobic conditions using GENbag anaer (BioMérieux), under microaerophilic conditions using GENbag microaer (BioMérieux) and in the presence of air, with or without 5%CO_2_. Growth was achieved anaerobically and weakly after 3 days under microaerophilic conditions, on blood-enriched Columbia agar and in TS broth medium. Growth on BHI agar medium, and on BHI agar medium supplemented with 1% NaCl was also weak, and occurred after 72h. Gram staining showed non spore-forming Gram-positive cocci (Figure 2). The motility test was negative. Cells grown anaerobically in TS broth medium have a mean diameter of 1.12 µm (min = 0.98µm; max = 1.33 µm), as determined using electron microscopic observation after negative staining with a 3% ammonium molybdate solution (Figure 3).

Strain 9402080^T^ exhibited catalase activity and no oxidase activity. Using an API 20A strip (BioMerieux, Marcy l’Etoile), positive reactions could be observed for D-Glucose, D-Lactose, D-Saccharose, D-Maltose, Salicin, D-Xylose, Gelatinase, Esculin, D-Mannose, and D-Trehalose. Using an API ZYM strip positive reactions were obtained for alkaline phosphatase (5nmol of hydrolyzed substrate), esterase (5nmol), esterase lipase (5nmol), leucine arylamidase (40nmol), acid phosphatase (5nmol), naphtophosphohydrolase (20nmol), and hyaluronidase (30nmol). Using an Api rapid id 32A, positive reactions could be observed for Arginine Dihydrolase, Beta Galactosidase, Beta Glucosidase, Beta Glucuronidase, N-Acetyl-beta-Glucosaminidase, Alpha-fucosidase, Mannose fermentation, Alkaline phosphatase, Arginine arylamidase, Leucine arylamidase, Pyroglutamate arylamidase, and Histidine arylamidase.

Regarding antibiotic susceptibility, *A. provenciensis* was susceptible to penicillin G, amoxicillin, cefotetan, imipenem, metronidazole and vancomycin. When compared to the representative species within the genus *Anaerococcus*, *A. provenciensis* exhibits the phenotypic characteristics details in Table 2.

**Table 2:** Differential characteristics of *Anaerococcus provenciensis* sp. nov., strain 9402080^T^, *A. octavius* strain NCTC 9810^T^, *A. prevotii* strain NCTC 11806^T^, and *A. tetradius* strain DSM 2951^T^

**Properties**	***A. provenciensis***	***A. octavius***	***A.prevotii***	***A. tetradius***
Cell diameter (µm)	0.98-1.33	0.7-0.9	0.6-1.5	0.5-1.8
Oxygen requirement	Anaerobic	Anaerobic	Anaerobic	Anaerobic
Gram stain	Positive	Positive	Positive	Positive
Optimal growth temperature	35-37°C	na	na	na
Habitat	Human	Human	Human	Human
				
Enzyme production				
Indole	-	-	-	-
Alkaline Phosphatase	+	-	-	-
Urease	-	-	+	+
Catalase	+	-	+	-
Gelatinase	+/-	na	na	na
Activity of				
Phosphatase	Acid phosphatase	na	na	na
	Naphtolphosphohydrolase			
Saccharolytic enzymes	ß-glucuronidase	-	α-glucosidase	α-glucosidase
			ß-glucuronidase	ß-glucosidase
				ß-glucuronidase
Proteolytic enzymes	Leucine arylamidase	Proline arylamidase	Arginine arylamidase	Arginine arylamidase
		Pyroglutamyl arylamidase	Pyroglutamyl arylamidase	Histidine arylamidase
			Histidine arylamidase	
Utilization of				
Glucose	+	+	-	+
Mannose	-	+	+	+
Lactose	+	-	-	-
Raffinose	-	-	+	+

**Figure 4:**
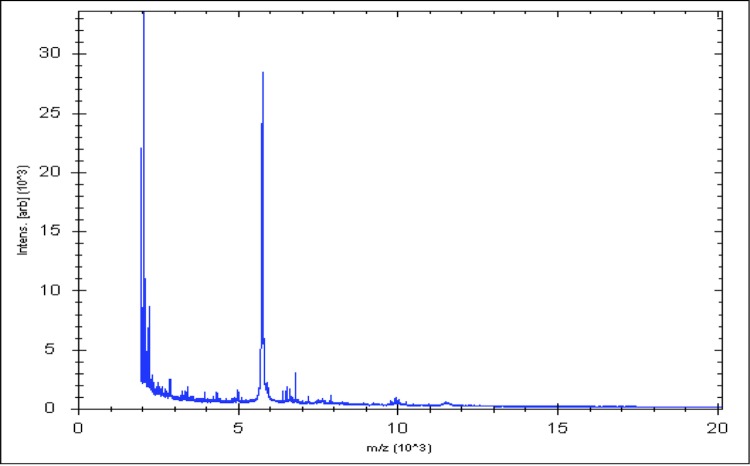
Reference mass spectrum from *A. provenciensis* strain 9402080^T^. Spectra from 10 individual colonies were compared and a reference spectrum was generated.

**Figure 5:**
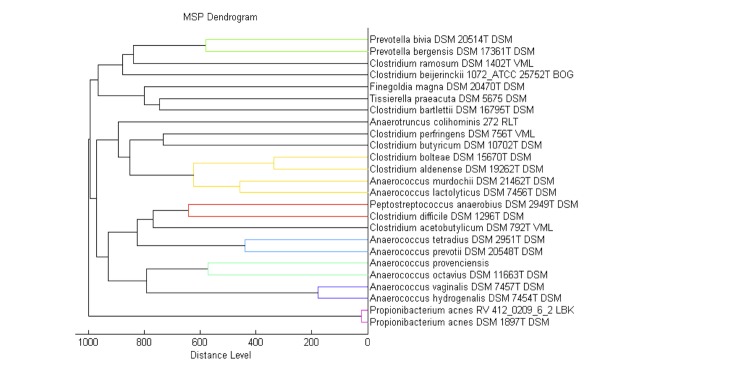
A dendrogram based on the comparison of the *A. provenciensis* strain 9402080^T^ MALDI-TOF reference spectrum with the spectra from 24 other species of the order of *Clostridiales*

Matrix-assisted laser-desorption/ionization time-of-flight (MALDI-TOF) MS protein analysis was carried out as previously described [36]. Briefly, a pipette tip was used to pick an isolated bacterial colony from a culture agar plate and spread it as a thin film on a MTP 384 MALDI-TOF target plate (Bruker Daltonics, Germany). Ten distinct deposits were done for strain *A. provenciensis* strain 9402080^T^, from ten isolated colonies. Each smear was overlaid with 2µL of matrix solution (saturated solution of alpha-cyano-4-hydroxycinnamic acid) in 50% acetonitrile, 2.5% tri-fluoracetic acid, and allowed to dry for five minutes. Measurements were performed with a Microflex spectrometer (Bruker). Spectra were recorded in the positive linear mode for the mass range of 2,000 to 20,000 Da (parameter settings: ion source 1 (ISI), 20kV; IS2, 18.5 kV; lens, 7 kV). A spectrum was obtained after 675 shots at a variable laser power. The time of acquisition was between 30 seconds and 1 minute per spot. The ten 9402080^T^ spectra were imported into the MALDI BioTyper software (version 2.0, Bruker) and analyzed by standard pattern matching (with default parameter settings) against the main spectra of 5,697 bacteria that were used as reference data in the BioTyper database. The method of identification includes the m/z from 3,000 to 15,000 Da. For every spectrum, 100 peaks at most were taken into account and compared with the spectra in database. A score enabled the presumptive identification and discrimination of the tested species from those in a database: a score > 2 with a validated species enabled the identification at the species level; a score > 1.7 but < 2 enabled the identification at the genus level; and a score < 1.7 did not enable any identification. For strain 9402080^T^, no significance score was obtained, thus suggesting that our isolate was not a member of a known species. We added the spectrum from strain 9402080^T^ (Figure 4) to our database. A dendrogram was constructed with the MALDI Bio Typer software (version 2.0, Bruker), comparing the reference spectrum of strain 9402080^T^ with reference spectra of 24 bacterial species, all belonging to the order of *Clostridiales*. In this dendrogram, strain 9402080^T^ appears on a separate branch within the genus *Anaerococcus* (Figure 5).

## Genome sequencing information

### Genome project history

The organism was selected for sequencing on the basis of its phylogenetic position and 16S rDNA similarity to other members of the *Anaerococcus* genus, and is part of a study for recovering and analyzing anaerobic bacteria from deep samples. It was the 8th genome of an *Anaerococcus* species and the first genome of *Anaerococcus provenciensis* sp. nov. The Genbank accession number is CAJU020000000 (CAJU020000001-CAJU020000026) and consists of 26 contigs. Table 3 shows the project information and its association with MIGS version 2.0 compliance [24].

**Table 3:** Project information

**MIGS ID**	**Property**	**Term**
MIGS-31	Finishing quality	High-quality draft
MIGS-28	Libraries used	Two 454 paired end 3-kb library
MIGS-29	Sequencing platforms	454 GS FLX+ Titanium
MIGS-31.2	Fold coverage	43.71
MIGS-30	Assemblers	Newbler version 2.8
MIGS-32	Gene calling method	Prodigal
	INSDC ID	PRJEB85
	Genbank ID	CAJU020000000
	Genbank Date of Release	May 28, 2013
	Project relevance	Study of the human gut microbiome

### Growth conditions and DNA isolation

*Anaerococcus provenciensis* sp. nov. strain 9402080T, CSUR P121 = DSM 26345 was grown anaerobically on blood agar medium at 37°C. 10 petri dishes were spread and resuspended in 3x100µl of G2 buffer. A first mechanical lysis was performed with glass powder on the Fastprep-24 device(Sample Preparation system) from MP Biomedicals, USA using 2x20 seconds pulses. DNA was then incubated with lysozyme (30 minutes at 37°C) and extracted through the BioRobot EZ 1 Advanced XL (Qiagen).The DNA was then concentrated and purified on a Qiamp kit (Qiagen). The yield and the concentration were measured by the Quant-it Picogreenkit (Invitrogen) on the Genios_Tecan fluorometer at 21.1ng/µl.

### Genome sequencing and assembly

Two paired end library were pyrosequenced on the 454 Roche Titanium. This project was loaded twice on a 1/4 region for the 3 kb insert libraries on PTP Picotiterplates. 5µg of DNA was mechanically fragmented on the Hydroshear device (Digilab, Holliston, MA,USA) with an enrichment size at 3-4kb. The DNA fragmentation was visualized through the Agilent 2100 BioAnalyzer on a DNA LabChip 7500 with an optimal size of 3.82 kb. The library was constructed according to the 454 Titanium paired end protocol supplied by the manufacturer. Circularization and nebulization were performed and generated a pattern with a maximum at 575 bp. After PCR amplification through 15 cycles followed by double size selection, the single stranded paired end libraries was then quantified on the Agilent 2100 BioAnalyzer on a RNA Pico 6000 LabChip at 135pg/µL. The library concentration equivalence was calculated at 4.31x10^08^ molecules/µL. The library was stored at -20°C until use.

The 3kb paired end library was clonally amplified with 0.5 and 1 cpb in 4 emPCR reactions per condition with the GS Titanium SV emPCR Kit (Lib-L) v2 .The yield of the emPCR was 5.56 and 9.79% respectively according to the quality expected by the range of 5 to 20% from the Roche procedure.

Two times 790,000 beads were loaded on the GS FLX Titanium PicoTiterPlates PTP Kit 70×75 and sequenced with the GS FLX Titanium Sequencing Kit XLR70.

The 454 sequencing generated 650,718 reads (104,82 Mb) assembled into contigs and scaffolds using Newbler version 2.8 (Roche) and Opera software v1.2 [37] combined with GapFiller V1.10 [38] and some finishing using CLC Genomics Workbench. Finally, the available genome consists of 8 scaffolds and 26 contigs, with a 43.71× coverage.

### Genome annotation

Non-coding genes and miscellaneous features were predicted using RNAmmer [39], ARAGORN [40], Rfam [41], and PFAM [42]. Open Reading Frames (ORFs) were predicted using Prodigal [43] with default parameters. The predicted ORFs were excluded if they spanned a sequencing gap region. The functional annotation was achieved using BLASTP [44] against the GenBank database [45] and the Clusters of Orthologous Groups (COG) database [46] [47].

## Genome properties

**Figure 6:**
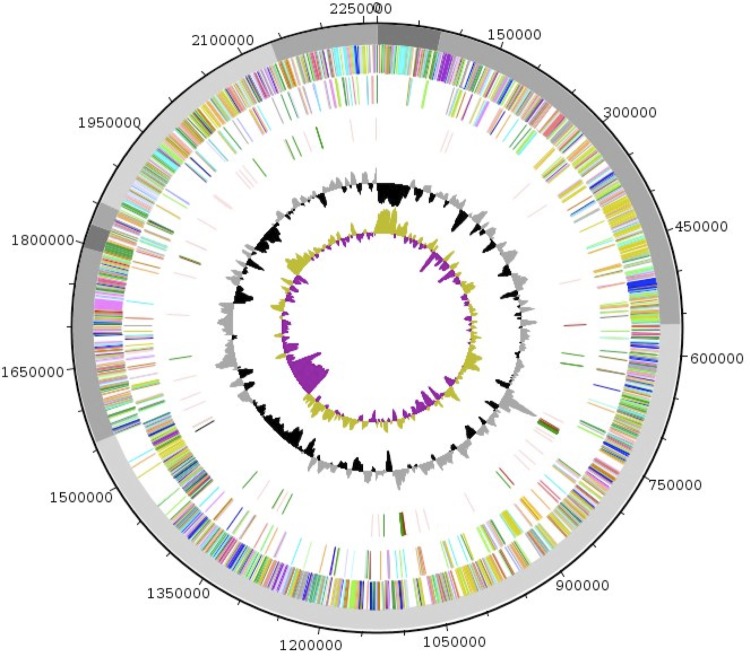
Graphical circular map of the genome. From outside to the center: scaffolds are in grey (unordered), genes on forward strand (colored by COG categories), genes on reverse strand (colored by COG categories), RNA genes (tRNAs green, rRNAs red, tm RNAs black, misc_RNA pink), GC content (black/grey), and GC skew (purple/olive).

The genome of *Anaerococcus provenciensis* strain 9402080^T^ is estimated to be 2.26 Mb long with a G+C content of 33.48% (Figure 5 and Table 4). A total of 2,099 protein-coding and 96 RNA genes, including 8 rRNA genes, 48 tRNA, 1 tmRNA and 39 miscellaneous other RNA were found. The majority of the protein-coding genes were assigned a putative function (74.8%); the remainder were annotated as hypothetical proteins. The distribution of genes into COGs functional categories is presented in Table 5 and Figure 6. The properties and the statistics of the genome are summarized in Tables 4 and 5.

**Table 4:** Nucleotide content and gene count levels of the genome

**Attribute**	**Value**	**% of total ^a^**
Genome size (bp)	2,265,283	100
DNA coding region (bp)	2,024,670	89.37
DNA G+C content (bp)	677.859	33.48
Total genes	2195	100
rRNA	8	0.36
tRNA	48	2.19
tmRNA	1	0.04
miscRNA	39	1.78
Protein-coding genes	2099	95.62
Genes with function prediction	1570	74.79
Genes assigned to COGs	2077	98.95

^a^ The total is based on either the size of the genome in base pairs or the total number of protein coding genes in the annotated genome

**Table 5:** Number of genes associated with the 25 general COG functional categories

**Code**	**Value**	**%age^a^**	**Description**
J	152	6.76	Translation
A	4	0.18	RNA processing and modification
K	174	7.74	Transcription
L	178	7.92	Replication, recombination and repair
B	4	0.18	Chromatin structure and dynamics
D	40	1.78	Cell cycle control, mitosis and meiosis
Y	0	0	Nuclear structure
V	92	4.09	Defense mechanisms
T	82	3.65	Signal transduction mechanisms
M	99	4.41	Cell wall/membrane biogenesis
N	14	0.62	Cell motility
Z	5	0.22	Cytoskeleton
W	0	0	Extracellular structures
U	51	2.27	Intracellular trafficking and secretion
O	78	3.48	Posttranslational modification, protein turnover, chaperones
C	130	5.78	Energy production and conversion
G	221	9.83	Carbohydrate transport and metabolism
E	125	5.56	Amino acid transport and metabolism
F	64	2.85	Nucleotide transport and metabolism
H	59	2.62	Coenzyme transport and metabolism
I	51	2.27	Lipid transport and metabolism
P	127	5.65	Inorganic ion transport and metabolism
Q	17	0.75	Secondary metabolites biosynthesis, transport and catabolism
R	238	10.6	General function prediction only
S	220	9.79	Function unknown
-	22	0.98	Not in COGs

^a^ The percentage is based on the total number of protein coding genes in the annotated genome.

## Insights into the genome sequence

We made some brief comparisons of *Anaerococcus provenciensis* against *Anaerococcus prevotii* DSM 20548 (NC_013171) which is currently the closest available genome. This genome contains 1 chromosome (accession number: NC_013171) and 1 plasmid (accession number: NC_013164).

The draft genome sequence of *Anaerococcus provenciensis* is bigger than that of *Anaerococcus prevotii* (2.26 Mbp and 1.99 Mbp, respectively). The G+C content (33.48%) is slightly lower than that of *Anaerococcus prevotii* (35.7%). *Anaerococcus provenciensis* has more coding-genes (2,099 predicted genes against 1,916 genes), but the ratios of the number of genes per Mbp genome size are relatively close (1079.22 – 962.81).

**Table 6:** Comparison of the percentage of genes associated with the 25 general COG functional categories for *Anaerococcus provenciensis* and *Anaerococcus prevotii* DSM 20548.

Code	*A. provenciensis* % of total	*A. prevotii* % of total	Difference (in %)	COG description
J	6.76	7.53	-0.77	Translation
A	0.18	0.10	0.08	RNA processing and modification
K	7.74	6.91	0.83	Transcription
L	7.92	6.13	1.79	Replication, recombination and repair
B	0.18	0.16	0.02	Chromatin structure and dynamics
D	1.78	1.56	0.22	Cell cycle control, mitosis and meiosis
Y	0	0.05	-0.05	Nuclear structure
V	4.09	3.43	0.66	Defense mechanisms
T	3.65	3.17	0.48	Signal transduction mechanisms
M	4.41	5.24	-0.83	Cell wall/membrane biogenesis
N	0.62	0.36	0.26	Cell motility
Z	0.22	0.16	0.06	Cytoskeleton
W	0	0	0	Extracellular structures
U	2.27	1.92	0.35	Intracellular trafficking and secretion
O	3.48	3.63	-0.15	Posttranslational modification, protein turnover, chaperones
C	5.78	6.59	-0.81	Energy production and conversion
G	9.83	8.41	1.42	Carbohydrate transport and metabolism
E	5.56	6.65	-1.09	Amino acid transport and metabolism
F	2.85	3.69	-0.84	Nucleotide transport and metabolism
H	2.62	3.58	-0.96	Coenzyme transport and metabolism
I	2.27	2.34	-0.07	Lipid transport and metabolism
P	5.65	6.80	-1.15	Inorganic ion transport and metabolism
Q	0.75	0.78	-0.03	Secondary metabolites biosynthesis, transport and catabolism
R	10.6	11.21	-0.61	General function prediction only
S	9.79	9.61	0.18	Function unknown
-	0.98	0.99	-0.01	Not in COGs

Table 6 presents the difference in gene numbers (in percentage) from each COG category between *Anaerococcus provenciensis* and *Anaerococcus prevotii* DSM 20548. The totals are highly similar in the two species. The biggest difference is in the COG "Carbohydrate Metabolism and transportation" category, which does not exceed 1.42%.

## Conclusion

On the basis of phenotypic, phylogenetic and genomic analysis, we formally propose the creation of *Anaerococcus provenciensis* sp. nov. that contains the strain 9402080^T^. This bacterium has been found in Marseille, France.

### Description of *Anaerococcus provenciensis* sp. nov.

*Anaerococcus provenciensis* (pro.ven.ci.en’cis; L. gen. masc. n. provenciensis, pertaining to Provence, the name of the aeae, south-east of France, where the type strain was isolated). Isolated from a cerebral abscess sample from a patient from Marseille. *A. provenciensis* is a Gram-positive cocci, obligately anaerobic, non-spore-forming bacterium. Grows at 37°C in anaerobic atmosphere. Negative for indole. Non-motile. The G+C content of the genome is 33.48%. The type strain is 9402080^T^(= CSUR P121 = DSM 26345).
